# Early detection of lung cancer in a real-world cohort *via* tumor-associated immune autoantibody and imaging combination

**DOI:** 10.3389/fonc.2023.1166894

**Published:** 2023-04-04

**Authors:** Zhong Liu, Feng Zhang, Jianwen Jiang, Chenzhao Zhao, Lu Zhu, Chenbing Liu, Nan Li, Lihong Qiu, Chao Shen, Di Sheng, Qiang Zeng

**Affiliations:** ^1^ Health Management Center, The First Affiliated Hospital of Zhejiang University School of Medicine, Hangzhou, China; ^2^ Department of Health Management Institute, The Second Medical Center & National Clinical Research Center for Geriatric Diseases, Chinese People’s Liberation Army (PLA) General Hospital, Beijing, China

**Keywords:** lung cancer, autoantibody, LDCT, immune, early detection

## Abstract

**Background:**

Efficient early detection methods for lung cancer can significantly decrease patient mortality. One promising approach is the use of tumor-associated autoantibodies (TAABs) as a diagnostic tool. In this study, the researchers aimed to evaluate the potential of seven TAABs in detecting lung cancer within a population undergoing routine health examinations. The results of this study could provide valuable insights into the utility of TAABs for lung cancer screening and diagnosis.

**Methods:**

In this study, the serum concentrations of specific antibodies were measured using enzyme-linked immunosorbent assay (ELISA) in a cohort of 15,430 subjects. The efficacy of both a 7-TAAB panel and LDCT for lung cancer detection were evaluated through receiver operating characteristic (ROC) analyses, with sensitivity, specificity, positive predictive value (PPV), and negative predictive value (NPV) being assessed and compared. These results could have significant implications for the development of improved screening methods for lung cancer.

**Results:**

Over the 12-month observation period, 26 individuals were diagnosed with lung cancer. The 7-TAAB panel demonstrated promising sensitivity (61.5%) and a high degree of specificity (88.5%). The panel’s area under the receiver operating characteristic (ROC) curve was 0.8062, which was superior to that of any individual TAAB. In stage I patients, the sensitivity of the panel was 50%. In our cohort, there was no gender or age bias observed. This 7-TAAB panel showed a sensitivity of approximately 60% in detecting lung cancer, regardless of histological subtype or lesion size. Notably, ground-glass nodules had a higher diagnostic rate than solid nodules (83.3% vs. 36.4%, P = 0.021). The ROC analyses further revealed that the combination of LDCT with the 7-TAAB assay exhibited a significantly superior diagnostic efficacy than LDCT alone.

**Conclusion:**

In the context of the study, it was demonstrated that the 7-TAAB panel showed improved detective efficacy of LDCT, thus serving as an effective aid for the detection of lung cancer in real-world scenarios.

## Introduction

In recent years, lung cancer has remained the leading cause of cancer-related morbidity and mortality globally (1). Early diagnosis of lung cancer is critical for patients to receive timely treatment and improve their quality of life. However, due to the insidious nature of the disease, most patients are already at an advanced stage upon initial diagnosis, leading to poor prognoses (2). While the 5-year survival rate for stage I lung cancer patients is around 75%, the rate drops to approximately 15% for patients with advanced lung cancer (3). Although progress in research and drug improved the treatment strategies against advanced lung cancer, it is still a disease with poor prognosis (4). Therefore, early detection is necessary for the patients to obtain timely treatments and improve life quality.

Currently, low-dose computed tomography (LDCT) is widely used for early detection of lung cancer. The National Lung Screening Trial (NLST) demonstrated that screening with LDCT in a high-risk population can reduce lung cancer mortality by 20% compared to chest X-rays (5). The NELSON trial also reported a 24% reduction in lung cancer mortality after 10 years of follow-up of 13131 males (6). However, the limitations of LDCT alone in early lung cancer screening, including overdiagnosis and resource constraints, cannot be ignored (7). Although LDCT is sensitive in detecting early lung lesions with a lower radiation dose, it could result in excessive medical treatments and unnecessary anxiety among patients (8).

Most previous studies utilizing LDCT for lung cancer screening focused on high-risk populations defined by smoking history or certain age. However, a real-world lung cancer detection study conducted by multiple centers in China found that more non-smokers were diagnosed with lung cancer than smokers, highlighting the importance of studies in real-world cohorts (9). As most individuals in China undergo regular health examinations, early detection of lung cancer should be considered in such examinations in the real-world setting (10).

Serum tumor-associated autoantibodies (TAABs) against tumor-associated autologous antigens have shown great promise as a biomarker for early detection of lung cancer (11). Studies have shown that the humoral immune system can recognize aberrant proteins derived from tumor cells at an early stage, leading to the production of a large number of autoantibodies, which are more readily detectable (12). Moreover, the detection of significantly increased TAABs can be conducted even before the formation of visible lesions through CT scans (13). As a liquid biopsy, the measurement of TAABs in peripheral blood is convenient and noninvasive, making it a potentially effective supplemental examination to LDCT in lung cancer detection (14). The National Health Service (NHS) found that TAABs could help identify those at high risk of lung cancer and reduce the incidence of advanced lung cancer at diagnosis (15). A study focusing on a panel of TAABs in European patients showed a specificity of 87% and a sensitivity of 41%, with a 5.4-fold increased risk of lung cancer in a positive result group (16). retrospective studies have demonstrated that TAABs have appropriate value in the detection of lung cancer in these populations.

In a recent development, a kit capable of detecting seven tumor-associated autoantibodies (7-TAAB) has been approved by the China Food and Drug Administration (CFDA) for the Chinese population (17). These autoantibodies include P53, GAGE7, PGP9.5, CAGE, MAGEA1, SOX2, and GBU4-5. As these autoantibodies are specific to lung cancer, they are expected to assist in its diagnosis. We aim to investigate the usefulness of TAABs in the real world cohort to see if they can improve the capabilities of low-dose computed tomography (LDCT) in lung cancer screening. To this end, we plan to conduct LDCT alongside the 7-TAAB assay on a population undergoing health examination and analyze their value in lung cancer detection.

## Material and methods

### Subjects and blood samples

In this study, a cohort of 15,430 individuals who underwent a health examination at The First Affiliated Hospital of Zhejiang University, School of Medicine between August 2019 and December 2021 were included. Eligibility for participation was based on the following criteria: 1) aged 18 years or older, 2) underwent LDCT in the health examination, and 3) completed the 7-TAAB assay during the health examination. Participants who met any of the following exclusion criteria were not included in the study: 1) individuals who had undergone chemotherapy, radiotherapy, targeted therapy, or surgical resection, 2) individuals who had a history of other malignancies, and 3) individuals taking immunity inhibitors.

Lung cancer was defined according to the World Health Organization Classification of Tumors (18). This investigation was approved by the Ethics Committee of The First Affiliated Hospital of Zhejiang University, School of Medicine. All subjects signed written informed consents. The serum were obtained from whole blood of the subjects by centrifuging at 3,000 g for 15 mins at 4°C, and were immediately analyzed by our assays.

### The TAAB assay and cut-off value

The ELISA kit (Cat. No. 20160501, 20160502) was kindly provided by CancerProbe biotechnology corporation (Hangzhou, China). All laboratory staff were blinded to the identities of the samples. The serum concentrations of the 7-TAAB were quantitated according to the instructions. The optical density (O.D.) was measured at 450 nm on a Dynex MRX Revelation microplate reader (Vienna, VA, USA). Each sample was tested in triplicate and preset commercial cut-off values were applied.

### LDCT inspection

A SIEMENS SOMATOM (Berlin, Germany) Definition flash spiral CT machine was used for low-dose lung cancer scanning. For patients with multiple nodules, the dominant one was selected for analysis. The CT diagnoses were reported by at least two experienced radiologists.

### Outcome criteria

The results of LDCT and 7-TAAB would be considered as true positive or negative if they were consistent with the histopathological diagnosis, otherwise, it would be considered as false positive or negative. In LDCT and 7-TAAB assay combined detection group, the result would be judged as positive when one of them was positive and as negative when both of them was negative. The definition of positive result of 7-TAAB assay was having at least one elevated TAAB signal in the panel.

### Evaluation of detecting methods

Histopathological result was regarded as the gold standard for lung cancer diagnosis. The results of detection were divided into true positive (a), false positive (b), false negative (c), and true negative (d). The calculation formular were sensitivity = a/(a + c), specificity = d/(d + b), consistency = (a + d)/(a + b + c + d), and positive predictive value (PPV) = a/(a + b), negative predictive value (NPV) = d/(d + c).

### Statistical analysis

The raw data were analyzed by Prism 6.0 (GraphPad software, La Jolla, CA, USA). The Wilcoxon rank sum test was used for comparison between the two groups. Count data were expressed as rates (%). AUCs were obtained according to ROC analysis, and the optimal critical values for the models in the detection of lung cancer were according to the Youden index. The sensitivity, specificity, consistency, PPV and NPV were statistically calculated using the four-table method. P < 0.05 was statistically significant.

## Results

### Study population

In our hospital, a total of 15,430 subjects were included in this real-world cohort, consisting of 9,062 males (58.7%) and 6,368 females (41.3%) who underwent health examination. Among them, 11,494 (74.5%) were non-smokers, while 3,936 (25.5%) were either smokers or had a history of smoking. **Table 1** summarizes the clinical characteristics of the subjects. The LDCT scans revealed that 7,898 (51.2%) subjects had at least one pulmonary nodule, and 2,059 (13.3%) subjects had a positive 7-TAAB response in the population. During the follow-up, 26 subjects were diagnosed with lung cancer by histopathological methods, while the others remained healthy or were diagnosed with benign diseases.

**Table 1:** Clinical characteristics of the physical examination population after a 12-month follow.

Characteristics	Lung cancer (n = 26)	Benign lung lesion (n = 7872)	Health controls (n =7532)
Age, years (mean, range)	55.85 (30–77)	52.07 (28-93)	48.76 (18-73)
Gender, n (%)			
Male	11 (42.3)	4737 (60.2)	4314 (57.3)
Female	15 (57.7)	3135 (39.8)	3218 (42.7)
Smoking, n (%)			
Never	18 (69.2)	5668 (72.0)	5808 (77.1)
Ever/current	8 (30.8)	2204 (28.0)	1724 (22.9)
Cancer subtype, n (%)			
Adenocarcinoma	23 (88.5)	/	/
Non-adenocarcinoma	3 (11.5)	/	/
Stage, n (%)			
I	8 (30.8)	/	/
II-IV	18 (69.2)	/	/
7-TAAB, n (%)			
Positive	16 (61.5)	1112 (14.1)	931 (12.4)
Negative	10 (38.5)	6760 (85.9)	6601 (87.6)
Density of nodules, n (%)			
Solid	12 (46.2)	4337 (55.1)	/
Ground glass	14 (53.8)	3535 (44.9)	/
Lesion size, n (%)			
φ ≤ 1 cm	11 (42.3)	5746 (72.9)	/
φ > 1 cm	15 (57.7)	2126 (27.1)	/

### Detection rates of pulmonary nodule by LDCT in the subjects of different genders and ages

In this cohort study, LDCT scans detected at least one nodule in 7898 cases, with a detection rate of 51.2%. The rates did not significantly differ between the male and female groups, but there was a significant age-related difference in nodule rates among the subjects (p<0.001), suggesting that individuals over the age of 40 are more likely to develop nodules (**Table 2**).

**Table 2:** Detection rates of pulmonary nodule by LDCT in the subjects of different genders and ages.

Groups	Subjects with pulmonary nodules, n (%)	χ^2^	P value
Age, years		1259	<0.001
< 40	711/3117 (22.8)		
≥40	7187/12313 (58.4)		
Gender		4.044	0.076
Male	4577/9062 (50.5)		
Female	3321/6368 (52.2)		

### The 7-TAAB levels in lung cancer patients and healthy controls

**Figure 1:**
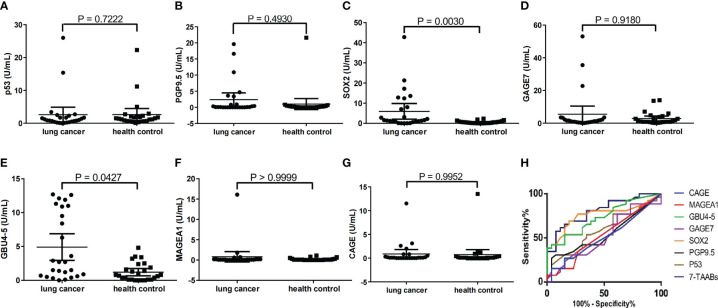
Reactivity of each TAAB in patients and matched healthy controls. **(A)** p53 **(B)** PGP9.5; **(C)** SOX2, **(D)** GAGE7; **(E)** GBU4-5; **(F)** MAGEA1; **(G)** CAGE. **(H)** AUCs for each single TAAB and the 7-TAAB panel in lung cancer patients versus healthy controls.

Serum samples from 26 lung cancer patients and 26 age-, gender-, and smoking history-matched healthy controls were analyzed for 7-TAAB levels. The positive rate of the complete panel of 7-TAAB (including P53, PGP9.5, SOX2, GAGE7, GBU4-5, MAGEA1, and CAGE) was found to be significantly higher in lung cancer patients than in controls (**Table 3**). Further analysis revealed that levels of SOX2 and GBU4-5 were specifically elevated in patients when compared to healthy controls (**Figures 1A–G**). The differential TAAB profiling of each case may provide insights into the underlying molecular mechanisms of carcinogenesis.

**Table 3:** The positive rates of TAABs in lung cancer patients and their age-, gender- and -smoking-history-matched healthy controls.

Characteristic	Lung cancer (%)	Health controls (%)
P53	7.70	3.85
PGP9.5	7.70	3.85
SOX2	23.10	0.00
GAGE7	11.50	0.00
GBU4-5	34.60	0.00
MAGEA1	3.85	0.00
CAGE	3.85	3.85
7-TAABs	61.50	11.50

### Comparison of the clinical characteristics of lung cancer patients with different 7-TAAB responses

In this study, the levels of 7-TAABs were analyzed in a cohort of lung cancer patients and healthy controls. The results revealed no significant difference in 7-TAABs levels between the groups based on gender (P = 0.530), age (P = 0.197), or lesion size (P = 0.768) (**Table 4**). Notably, the 7-TAAB panel showed promise in early detection of lung cancer, with a sensitivity of 50.0% for stage I subjects. When stratified by histopathological types, the majority of diagnosed cases (23 out of 52) were adenocarcinoma, and the 7-TAAB assay had a sensitivity of 60.9% in this group. The remaining cases were non-adenocarcinoma, and the assay had a sensitivity of 66.7% in this group. Importantly, there was no significant preference of the assay for either histopathological type (P = 0.846). The assay also had a higher diagnosis rate for ground glass nodules (83.3%) than solid ones (36.4%) (P = 0.021), suggesting its potential for aiding in the quality judgment of nodules (**Table 4**).

**Table 4:** Comparison of the clinical characteristics of the lung cancer patients with different 7-TAAB responses.

Characteristics	7-TAABs positive cases, n (%)	7-TAABs negative cases, n (%)	χ^2^	P value
Age			1.664	0.197
< 40	0/1 (0.0)	1/1 (100.0)		
≥40	16/25 (64.0)	9/25 (36.0)		
Gender			0.394	0.530
Male	6/11 (54.5)	5/11 (45.5)		
Female	10/15 (66.7)	5/15 (33.3)		
Density of nodules			5.316	0.021
Solid	4/11 (36.4)	7/11 (63.6)		
Ground glass	10/12 (83.3)	2/12 (16.7)		
Lesion size			0.087	0.768
φ ≤ 1 cm	6/10 (60.0)	4/10 (40.0)		
φ > 1 cm	7/13 (53.8)	6/13 (46.2)		
Stage			0.650	0.420
I	4/8 (50.0)	4/8 (50.0)		
II-IV	12/18 (66.7)	6/18 (33.3)		
Subtypes			0.038	0.846
Adenocarcinoma	14/23 (60.9)	9/23 (39.1)		
Non-adenocarcinoma	2/3 (66.7)	1/3 (33.3)		

### The clinical value of each detecting method for lung cancer

The sensitivities of each TAAB in the panel for detecting lung cancer varied greatly, ranging from 3.85% to 34.6%. Notably, GBU4-5 was found to be the most sensitive, with a positive rate of 34.6% in diagnosed patients, while MAGEA1 and CAGE exhibited the lowest sensitivities at 3.85%. The patient group showed specific positivity for SOX2, GAGE7, GBU4-5, and MAGEA1, while P53, PGP9.5, and CAGE sporadically showed positivity in the healthy controls (**Table 3**, **Figure 1**).

The 7-TAAB assay, which defined positivity as any TAAB in the panel showing an increased level according to the cut-offs, demonstrated a sensitivity of 61.5% and a specificity of 88.5% in detecting lung cancer. Moreover, the ROC analyses revealed that the 7-TAAB assay was more efficacious than any single TAAB assay based on the AUCs (**Figure 1H**), with an improved AUC of 0.8062 in the cohort. The panel’s curve had a sensitivity of 65.4% and a specificity of 84.6% based on the Youden index, while the PPV and NPV were 84.2% and 69.7%, respectively. In contrast, LDCT exhibited higher sensitivity (88.5%) but much lower specificity (46.2%) in the same cohort. Due to the high false positive rate, LDCT had a PPV of 62.2% and a consistency with histopathology of 67.3% (**Table 5**).

**Table 5:** Comparison of LDCT, 7-TAABs assay, and the combined model for the detection of lung cancer.

Methods	Sensitivity (%)	Specificity (%)	Consistency (%)	PPV (%)	NPV (%)
LDCT	88.5	46.2	67.3	62.2	80.0
7-TAABs assay	61.5	88.5	75.0	84.2	69.7
LDCT+7-TAABs assay	96.1	42.3	69.2	62.5	91.7

**Figure 2:**
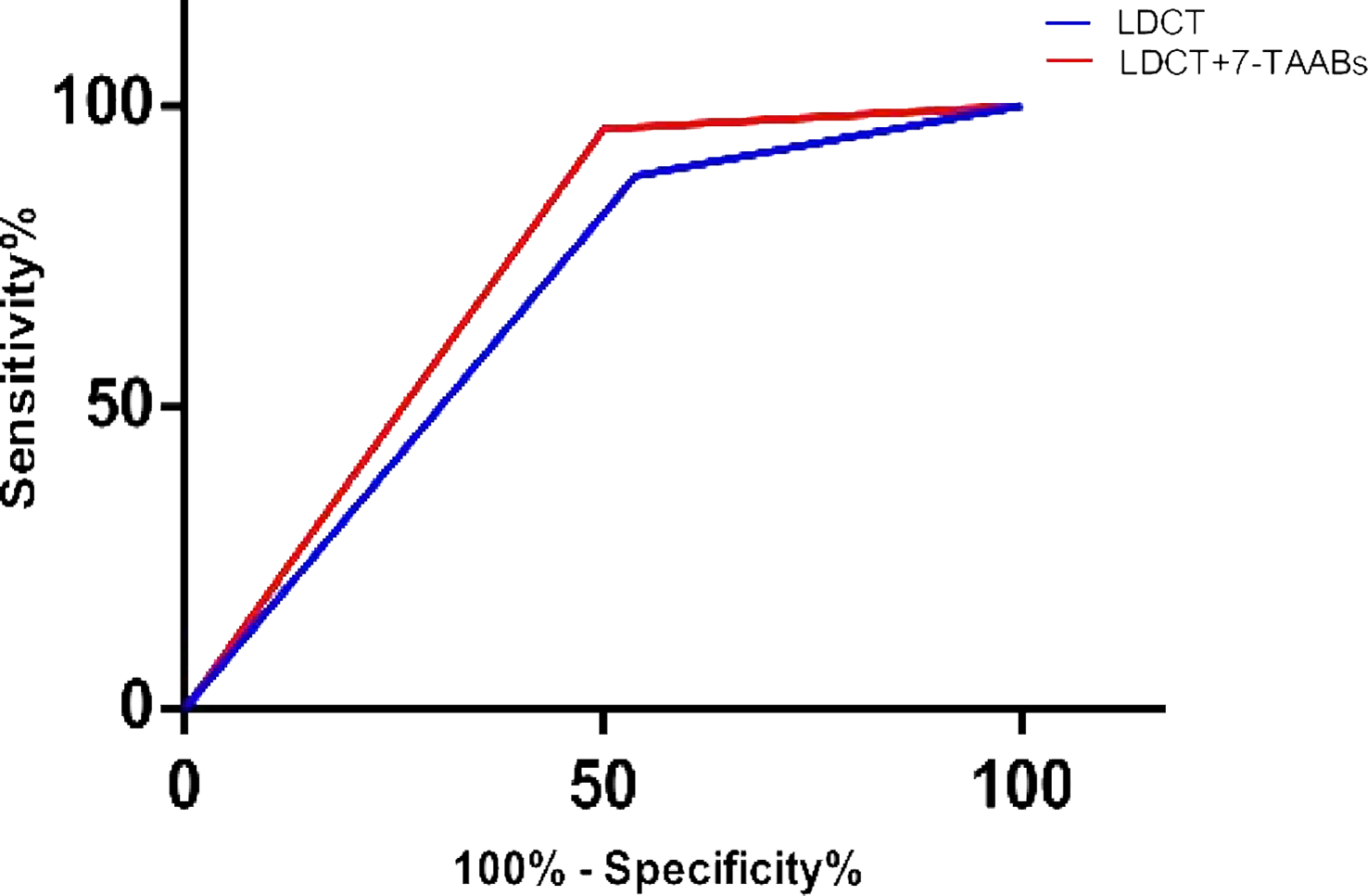
AUCs for LDCT and the combined detection in patients versus healthy controls.

However, combining the 7-TAAB assay and LDCT as an evaluation model for lung cancer detection significantly improved sensitivity (96.1%) and NPV (91.7%), while maintaining the specificity close to LDCT’s. Moreover, the combined model’s AUC (0.7308, 95% CI: 0.590-0.871, P = 0.004) was better than that of the routinely used LDCT (AUC = 0.673, 95% CI: 0.524-0.822, P = 0.032) in detecting lung cancer (**Figure 2**).

## Discussion

In the pursuit of improving the prognosis and survival rates of lung cancer, early treatments have played a crucial role (19). The screening method of low-dose computed tomography (LDCT) has shown promise in reducing mortality rates for non-small cell lung cancer (NSCLC) by 20%, surpassing the effectiveness of routine chest X-rays (20). However, LDCT has its limitations in distinguishing between benign and malignant nodules, leading to a high rate of false-positive results and excessive treatments. Recent studies by Gao et al. have suggested that LDCT may contribute to the over-diagnosis of early-stage lung cancer, without improving mortality rates or the overall quality of patient survival (21). To address this issue, liquid biopsy has emerged as a promising technique for distinguishing invasive tumors from indolent ones (22). With liquid biopsy, physicians are able to gather more comprehensive biological information about lesions, which can inform more effective treatment options. In particular, the presence of positive autoantibodies in a patient’s blood has been associated with a significantly lower 5-year survival rate of 7.6% (23). Clinical biomarkers for lung cancer are typically linked to factors such as tumor stage, histopathological subtype, and tumor burden (24). However, the detection of tumor-associated autoantibodies (TAABs) through liquid biopsy has shown potential as an early indicator of lung cancer, as humoral immunity often responds to tumor-associated antigens months or even years before clinical manifestations become evident (25). Liquid biopsy is a less invasive and more cost-effective alternative to traditional biopsy methods, and may hold great promise for improving lung cancer diagnosis and treatment.

Within the panel of seven molecules examined, P53 exerts an inhibitory effect on tumor cell proliferation by participating in DNA repair pathways (26). PGP9.5, a biomarker specific to non-small cell lung cancer (NSCLC), functions as a ubiquitin hydrolase (27). SOX2 acts as a transcription factor in cells and serves as an independent prognostic indicator of poor outcome in lung adenocarcinoma (28). GAGE7 contains an antigenic peptide, the antibody levels of which are elevated in melanoma patients, and high levels of GAGE7 are associated with a poor prognosis (29). The ATP-binding RNA helicase GBU4-5 is an essential factor in tumorigenesis (30). MAGEA1, an antigen present in melanoma, is correlated with a poor prognosis in NSCLC (31). Furthermore, increased levels of CAGE have been detected in tumors of the liver, lung, and cervix (32).

Numerous studies worldwide have been conducted in the development of lung cancer detection tests, including the PAULA’s (Protein Assay Using Lung Cancer Analytes) test that utilized three tumor biomarkers, CEA, CA-125, and CYFRA 21-1, in combination with NY-ESO-1 to form a panel with a sensitivity of 77% and specificity of 80% for NSCLC patients (33). Another detection model for lung cancer, the EarlyCDT^®^-Lung, consisted of two sub-panels. The first sub-panel comprised six autoantibodies (P53, NY-ESO-1, CAGE, GBU4-5, Annexin I, and SOX2) and demonstrated a sensitivity/specificity of 46%/83%, while the second sub-panel contained seven candidates (P53, NY-ESO-1, CAGE, GBU4-5, SOX2, HuD, and MAGE A4) with a sensitivity/specificity of 37%/91%. Consistent with these observations, our panel exhibited good sensitivity and specificity in a real-world cohort that was independent of histological subtype and clinical stage.

The 7-TAAB assay kit developed by CancerProbe biotechnology corporation was utilized in this study to assess the detection capability of the panel in lung cancer patients, including those with different clinical stages and histological subtypes. Our results indicated that the 7-TAAB assay had comparable efficacy for patients at stages I and II-IV, providing greater benefits to asymptomatic patients at an early stage during health examination, regardless of gender or age. When considering lesion size and subtype, this panel effectively distinguished malignant nodules in patients with a sensitivity of approximately 60%. In addition, positive 7-TAAB results were more frequently detected in ground-glass nodules, a common type of nodule in outpatient settings, compared to solid nodules. These findings demonstrate the clinical value of the 7-TAAB assay in the health examination population, especially in combination with LDCT, which significantly improved the efficacy of LDCT in detecting lung cancer. However, further validation through multi-center studies is still necessary.

Several limitations of our research should be noted. Firstly, the follow-up data were insufficient to analyze the prognostic value of the panel due to the majority of subjects being diagnosed within the past year. Secondly, most subjects were from the southeast region of China. It is uncommon in other parts of the world to perform low dose CT on healthy adults as young as 18 years old with no known risk factors for lung cancer, such as smoking. Thirdly, the levels of 7-TAAB in other pathological conditions, such as interstitial lung disease, chronic obstructive pulmonary disease (COPD), or asthma, should be considered when distinguishing lung cancer from these conditions (34, 35). We acknowledge that the sample size of 26 patients in the control group is relatively small, and samples from the control group were collected according to the 1:1 design compared with the typical design (4:1). To improve the diagnostic capability of the assay, future studies may include joint analysis with other possible serum biomarkers, such as miRNA, ctDNA, or DNA methylation. Additionally, the molecular mechanism underlying the elevation of antibodies in lung cancer should be investigated in future research (36).

## Conclusion

To summarize, the 7-TAAB panel has demonstrated its potential as a powerful diagnostic tool for lung cancer detection in a real-world cohort, particularly when combined with LDCT. Gender and age did not appear to have any significant impact on the performance of the assay, and it showed a greater sensitivity for detecting ground-glass nodules. Our findings indicate that the combination of the 7-TAAB panel with LDCT can enhance sensitivity to 96.1% and NPV to 91.7%, thereby reducing the number of false positives generated by LDCT. Overall, our study highlights the clinical utility of the 7-TAAB panel in facilitating early detection of lung cancer, and may have significant implications for improving patient outcomes in this population.

## Data availability statement

The raw data supporting the conclusions of this article will be made available by the authors, without undue reservation.

## Ethics statement

The studies involving human participants were reviewed and approved by the Ethics Committee of the First Affiliated Hospital, College of Medicine, Zhejiang University.

## Author contributions

Conceptualization, ZL and QZ; methodology, JJ; software, CZ; formal analysis, LZ; investigation, CL; resources, NL; data curation, LQ; writing—original draft preparation, ZL; writ-ing—review and editing, CS; visualization, DS; supervision, QZ. All authors contributed to the article and approved the submitted version.
